# 

**Published:** 1845-12-01

**Authors:** 


BUFFALO

### MEDICAL JOURNAL.

EDITED BY

AUSTIN FLINT, M. D.

**Vol. 1. BUFFALO, DECEMBER, 1845. No. 7.**

### CONTENTS:

### ORIGINAL COMMUNICATIONS.

Notes of an European Tour. By F. H. HAMILTON, M. D., Prof. of Surgery in Geneva Medical College. P. 145
Rupture of an Ovarian Cyst by a fall; escape of its contents into peritoneal cavity; absorption, and radical cure. By JAMES P. WHITE, M. D. - - - - - - - - - - - - - - - 151
Case of Omental Hernia; removal of the Sac, with spermatic cord, and testicle. By ALDEN S. SPRAGUE, M. D. - - - - - - - - - - - - - - - - - - - 153
Case of Typhoid Fever, with Autopsy and remarks by G. N. BURWELL, M. D. - - - - - - 156
Letter from Accoucheur. No. 3. - - - - - - - - - - - - - - - - - 160

### EDITORIAL, MEDICAL INTELLIGENCE, ETC.

Physicians in Buffalo. - - - - - - - - - - - - - - - - - - - - 162
Treatment of Aneurism by compression. - - - - - - - - - - - - - - - 163
Electro Physiology. - - - - - - - - - - - - - - - - - - - - 164
Case of Placenta Praevia, treated by Prof. Simpson's method. - - - - - - - - - 166
Our Exchange Journals. - - - - - - - - - - - - - - - - - - 166
Medical Remembrancer, or Book of Emergencies. - - - - - - - - - - - - 167
Monograph on Strabismus, by F. H. Hamilton, M. D. - - - - - - - - - - - 167
Treatise on Hare lip, by Dr. S. P. Hullihen. - - - - - - - - - - - - - 167
Injury of head, &c., operation of trephining, by D. Brainard, M. D. - - - - - - - 168
Medical Fees, No. 3. - - - - - - - - - - - - - - - - - - - 168
Medical Legislation. - - - - - - - - - - - - - - - - - - - 168

BUFFALO:

C. F. S. THOMAS, PRINTER AND PUBLISHER,

Exchange Buildings, Third Story, Main Street.

